# Patterns of the low‐dose dexamethasone suppression test in canine hyperadrenocorticism revisited

**DOI:** 10.1111/vcp.12958

**Published:** 2021-03-16

**Authors:** Florian K. Zeugswetter, Alejandra Carranza Valencia, Kerstin Glavassevich, Ilse Schwendenwein

**Affiliations:** ^1^ Clinical Department for Small Animals and Horses University of Veterinary Medicine Vienna Vienna Austria; ^2^ Department of Pathobiology Central Laboratory University of Veterinary Medicine Vienna Vienna Austria

**Keywords:** cortisol, diagnostic test, dogs, increasing pattern, inverse pattern, likelihood ratio

## Abstract

**Background:**

The low‐dose dexamethasone suppression test (LDDT) is considered an accurate screening and valuable differentiation test in dogs with suspected hyperadrenocorticism (HAC). A recent study showed that the different response patterns not only provide complementary information about etiology, but also the probability of HAC in these patients.

**Objectives:**

We aimed to determine the diagnostic test performance of LDDT response patterns in a population of dogs from an animal hospital.

**Methods:**

The electronic database was retrospectively searched for dogs suspected of HAC that were given an LDDT. Dogs with acute non‐adrenal illnesses during the test were excluded. Response patterns were classified as complete suppression, lack of suppression, partial suppression, escape, inverse, and increasing patterns. Cortisol concentrations ≥ 27.59 nmol/L (≥1 µg/dL) 8 hours after dexamethasone administration were considered positive results irrespective of the patterns observed. Calculations included likelihood ratios (LRs) and predictive values (PVs).

**Results:**

HAC and non‐adrenal illness were diagnosed in 115 (54%) and 62 (46%) dogs, respectively. The positive (+) LRs (95% CI) for the lack of suppression, partial suppression, escape, and an inverse pattern to diagnose HAC were infinite, 8.09 (2‐32.72), 3.23 (0.75‐14), and 0.2 (0.06‐0.73), respectively.

**Conclusions:**

The study confirms that the “lack of suppression” pattern strongly supports a diagnosis of HAC. It shows that the “partial suppression” pattern moderately increases, and the “inverse” pattern decreases the likelihood of HAC. The fact that the study found no association between the “escape” pattern and a diagnosis of HAC, does not support its integration into decision making.

## 1 INTRODUCTION

In 2013, the American College of Veterinary Internal Medicine (ACVIM) published a consensus statement on the diagnosis of spontaneous hyperadrenocorticism (HAC) in dogs.^1^ The low‐dose dexamethasone suppression test (LDDT) was propagated as the screening test of choice with a recommendation to re‐evaluate the cutoff values. Further endocrine testing was strongly recommended for dogs with an “inverse” LDDT pattern, that is, a cortisol concentration > 27.59 nmol/L (>1 µg/dL) 4 hours (t_4_), and < 27.59 nmol/L (<1 µg/dL) at 8 hours (t_8_) after dexamethason administration, which was traditionally interpreted as a negative test result.

Mueller et al were the first to describe this pattern in five dogs with pituitary‐dependent hyperadrenocorticism (PDH).^2^ The authors hypothesized that it could reflect a new type of HAC, but also detected this pattern in two of 29 dogs with initially suspected, but later excluded, HAC.^2^ Five different LDDT patterns and the respective positive predictive values (PPV) to diagnose HAC were investigated in a subsequent study.^3^ The patterns were defined as complete suppression (t_4_ and t_8_ < 27.59 nmol/L [<1 µg/dL]), lack of suppression (t_4_ and t_8_ > 27.59 nmol/L [>1 µg/dL] and > 50% t_0_), partial suppression (t_4_ and t_8_ > 27.59 nmol/L [>1 µg/dL] and either or both < 50% t_0_), escape (t_4_ < 27.59 nmol/L [<1 µg/dL] and t_8_ > 27.59 nmol/L [>1 µg/dL]), and inverse (t_4_ > 27.59 nmol/L [>1 µg/dL] and t_8_ < 27.59 nmol/L [<1 µg/dL]). The “inverse” and the “escape” patterns had very low PPVs, and the authors raised a concern that these patterns might not be supportive of HAC. The “escape” pattern is currently considered a classic and common pattern of HAC.^4^ Additionally an “increasing” pattern, defined by a >50% increase in cortisol concentrations between any time point, was found to be potentially useful for differentiating pituitary‐dependent (PDH) and adrenal tumor hyperadrenocorticism (ATH).^3^

The differentiation between HAC subtypes is important, as the choice of treatment and prognosis could differ significantly.^1^ Using currently established criteria, approximately 60% of the dogs with HAC can be identified as having PDH with the LDDT alone.^4^ For dogs without a suppression pattern, additional tests, such as high‐dose dexamethasone tests, endogenous adrenocorticotropic hormone (ACTH) measurements, or diagnostic (adrenal or pituary) imaging, are necessary. Bennaim et al described an “increasing” pattern in 6 of 31 dogs without suppression, all of which were diagnosed with PDH.^3^ The authors speculated that transient increases could be a consequence of ACTH stimulation, not expected in dogs with ATH, and recommended additional studies be performed, including the use of more dogs.

The unexpected poor performance of the “escape” pattern in the study by Bennaim et al and the presentation of the new “increasing” pattern for differentiation,^3^ prompted us to perform this retrospective study. The primary aim was to investigate the diagnostic performance, primarily looking at the likelihood ratios (LRs) of various LDDT patterns to diagnose HAC in a population of dogs with suspected HAC. The hypothesis was that individual LDDT patterns, especially the lack of suppression and partial suppression patterns, have a high likelihood of diagnosing HAC and, thus, providing additional diagnostic support.

## 2 MATERIALS AND METHODS

### 2.1 Case selection and data collection

The electronic database (TIS VetWare, Agfa HealthCare) of the University of Veterinary Medicine Vienna/Austria was retrospectively searched for all dogs that underwent a LDDT between April 2001 to January 2019. The urinary corticoid to creatinine ratio (UCCR) was used as the first screening test, followed by the LDDT and the ACTH‐stimulation test. All tests were generally recommended, as no test has 100% diagnostic accuracy and a pre‐mortem gold standard does not exist.^1^

**FIGURE 1:**
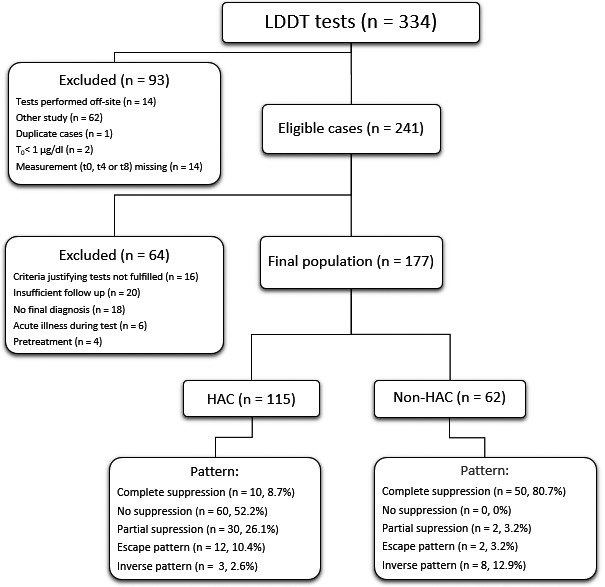
A flow chart depicting the selection of cases included in the study, the assignment of the cases to two groups, and the specific low‐dose dexamethasone suppression patterns, respectively. Abbreviations: HAC, hyperadrenocorticism; LDDT, Low‐dose dexamethasone test

Dogs were considered eligible for the study if the LDDT was performed on‐site with cortisol measurements reported at t_4_ and t_8_ after dexamethasone administration, and if basal cortisol concentrations (t_0_) were ≥27.59 nmol/L (≥1 µg/dL) (Figure 1). In cases with duplicate tests performed in one animal, only the first test results were included. The medical records were then retrospectively reviewed, and the patients were considered for inclusion if they showed at least one of the following common clinical signs: polyuria/polydipsia, polyphagia, alopecia, excessive panting, muscle weakness, abdominal distension; or two of the following uncommon clinical signs consistent with HAC, lethargy, skin hyperpigmentation or atrophy, comedones, poor hair regrowth, insulin‐resistant diabetes mellitus, pseudomyotonia; or one uncommon clinical sign of HAC and increased alkaline phosphatase activity (ALP); or after the identification of an adrenal mass or calcinosis cutis.^1^ Dogs with acute non‐adrenal illnesses (eg fever, icterus, pain, vomiting, diarrhea, untreated diabetes mellitus, or renal failure), leukocytoses with a leftshift, and dogs pre‐treated or treated with trilostane, ketoconazole, mitotane, glucocorticoids, or gestagens were excluded. Between the years 2004 to 2006, desmopressin stimulation tests^5^ were requested under the name LDDT and specifically marked. These tests were also excluded. Dogs with neurologic signs including inappetence and anorexia possibly caused by rapid growth or large pituitary tumors were included. The following information was retrieved for each dog whenever available: signalment (age, weight, gender, breed), common and uncommon clinical signs (yes/no), ALP activities, endocrine test results (UCCR, LDDT, high‐dose dexamethasone test, ACTH‐stimulation test, endogenous ACTH, heat stabile alkaline phosphatase activity [HS‐ALP]), adrenal ultrasonography or advanced imaging results, urine specific gravity, histopathology or postmortem examination results, alternative diagnoses, and trilostane treatment or adrenalectomy responses.

Cases were classified as having HAC if the owners reported a response to HAC treatments (ie, trilostane, ketoconazole, or adrenalectomy), or if postmortem examinations identified a pituitary adenoma or adrenocortical neoplasia with contralateral atrophy. Dogs were assigned to the non‐HAC group if an alternative diagnosis was made, if clinical signs did not progress or resolved without treatment, if the UCCR was negative during a later examination, and if pituitary adenoma or adrenocortical neoplasia was not detected at the postmortem examination. HAC cases were defined as PDH if endogenous ACTH was detectable, if ultrasonographic examination showed normal to symmetrical or mildly asymmetrical bilateral enlarged adrenal glands, and/or if magnetic resonance imaging (MRI) or computed tomography (CT) showed an enlargement of the pituitary gland (pituitary height /brain area ratio > 0.31).^6^, ^7^, ^8^, ^9^ Cases in the HAC group were assigned to the ATH‐subgroup if histopathologic or postmortem examinations revealed adrenocortical tumors and/or ultrasonographic examination showed unilateral nodular enlargement with vascular invasion or contralateral atrophy (≤5 mm).^10^

### 2.2 Endocrine tests and assays

**FIGURE 2:**
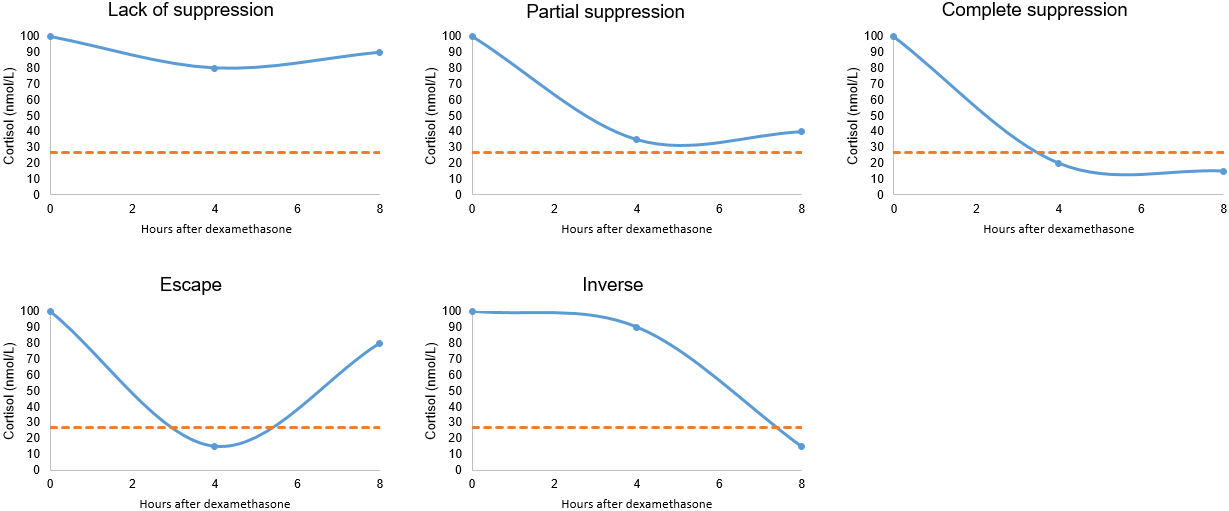
Suppression patterns of the low‐dose dexamethasone suppression test including lack of suppression (t_4_ and t_8_ ≥ 27.59 nmol/L, and >50% of t_0_ cortisol concentration), partial suppression (t_4_ and t_8_ ≥ 27.59 nmol/L, but either one or both <50% of t_0_ cortisol concentration), complete suppression (t_4_ and t_8_ < 27.59 nmol/L), and escape (t_4_ < 27.59 nmol/L and t_8_ ≥ 27.59 nmol/L), and inverse patterns (t_4_ ≥ 27.59 nmol/L and t_8_ < 27.59 nmol/L). Traditionally, a cortisol concentration ≥ 27.59 nmol/L at t_8_ is considered a positive test irrespective of the suppression pattern observed. 27.59 nmol/L = 1 µg/dL = 10 ng/mL

For UCCR determinations, urine was collected at home, preferably, but not necessarily, in the morning. UCCRs < 26.5 × 10^−6^, between 26.5 and 161.2 × 10^−6^, and > 161.2 × 10^−6^ were considered negative, suspect, or supportive for HAC, respectively.^11^ For LDDT‐testing, serum samples were stored and used within 18 hours after storage at 4°C. The testing was performed by measuring serum cortisol concentrations immediately before (t_0_), and 4 (t_4_) and 8 (t_8_) hours after intravenous administration of 0.01 mg/kg body mass dexamethasone (Dexamethason Nycomed 4 mg, Takeda Pharma).^1^ The dogs were not fasted, and were hospitalized only if the dog owners considered ambulatory testing more stressful for their animals. Other procedures, such as ultrasonography, were not permitted during testing. Adapted from an earlier study, each LDDT was then classified as complete suppression (t_4_ and t_8_ < 27.59 nmol/L [<1 mg/dL]), lack of suppression (t_4_ and t_8_ ≥ 27.59 nmol/L [≥1 mg/dL] and > 50% of the t_0_ cortisol concentration), partial suppression (t_4_ and t_8_ ≥ 27.59 nmol/L [≥1 mg/dL], but either one or both < 50% of the t_0_ cortisol concentration), escape pattern (t_4_ < 27.59 nmol/L [<1 mg/dL] and t_8_ ≥ 27.59 nmol/L [≥1 mg/dL]), and inverse pattern (t_4_ ≥ 27.59 nmol/L [≥1 mg/dL] and t_8_ < 27.59 nmol/L [<1 mg/dL]; Figure 2).^3^ A cortisol concentration ≥ 27.59 nmol/L [≥1 mg/dL] at t_8_ was considered a positive test irrespective of the suppression pattern observed. Additionally, the “increasing” pattern, potentially useful to differentiate between PDH and ATH and defined as an increase in the cortisol concentration of >50% between any time points in dogs with‐lack of suppression,^3^ was investigated.

For ACTH‐stimulation testing, serum cortisol concentrations were measured 60 minutes after intravenous administration of 125 µg (dogs ≤ 5 kg) or 250 µg (dogs > 5 kg) synthetic ACTH (Tetracosactide, Synacthen, Novartis Pharma, Vienna, Austria). A post‐ACTH concentration ≥ 579 nmol/L (≥21 µg/dl) was interpreted as a positive result.

Cortisol was measured using a competitive chemiluminescent immunoassay (Immulite 1000 and Immulite 2000xpi Cortisol, respectively, before and after 2017; Siemens Healthcare Diagnostics) validated for use in dogs.^12^, ^13^

Endogenous ACTH was measured in EDTA‐plasma using the Immulite 1000 and Immulite 2000 xpi ACTH assay (Siemens Healtcare Diagnostics) before and after 2017, respectively. An ACTH concentration in dogs with confirmed HAC of ≥2.2 pmol/L and later ≥1.1 pmol/L was considered diagnostic for PDH.^6^, ^7^

Total ALP and HS‐ALP activities were analyzed in lithium‐heparin plasma. Total ALP was measured using the Cobas ALP2‐assay on a Roche/Hitachi Cobas c502 analyzer (Roche Diagnostics), and HS‐ALP was assessed using the heat stability method at 65°C.

### 2.3 Statistical analysis

Distribution of the data was assessed with the Kolmogorov‐Smirnov test and data were given as the median and range. The sex distribution and frequency of clinical signs in the groups were compared using the chi‐square or Fisher exact test. The Mann‐Whitney *U* test was used for age, weight, and biochemical test results. The cutoff values associated with the highest sensitivities and specificities were determined using receiver operating characteristic (ROC) curve analyses and the calculation of differential positive rates. Analyses further included the calculation of the positive and negative LRs (+LR, −LR), as well as the positive and negative predictive values (PPVs, NPVs) of the different LDDT patterns and the LDDT in general (with and without including the inverse pattern as a positive test). The online statistical software (MEDCALC, https://www.medcalc.org/calc/diagnostic_test.php, accessed 18.03.20) was used for all calculations. All other analyses were performed using IBM SPSS v. 24 (IBM Corporation), and a *P*‐value <.05 was considered significant.

## 3 RESULTS

### 3.1 Study population

Three‐hundred and thirty‐four LDDT test results were retrieved. Thirty‐one tests were excluded due to off‐site testing (n = 14), incomplete data (t_0_, t_4_, or t_8_ missing, n = 14), duplicate testing (n = 1), and t_0_ < 27.59 nmol/L (<1 µg/dL; n = 2). Sixty‐two tests were excluded because the desmopressin stimulation test had been requested under this request tag and specifically marked. Of the eligible 241 tests, 64 were excluded after review of the medical records (Figure 1). The study population finally consisted of 177 patients (HAC = 115, non‐HAC = 62 dogs), resulting in a prevalence of HAC of 65%. Ninety‐two (80%), 19 (17%), and 3 (2%) dogs were diagnosed with PDH, ATH, and a combination of both PDH and ATH, respectively. No differential diagnoses were possible in one dog (1%).

The median (range) age at diagnosis was 11 (0.92 to 16.25) and 11.8 (3.7 to 15.4) years in the HAC and non‐HAC groups, respectively (*P* =.552). The HAC group included 29 (25.2%) intact male, 22 (19.1%) neutered male, 14 (12.2%) intact female, and 48 (41.7%) neutered female dogs. The non‐HAC group included 15 (24.2%) intact male, 15 (24.2%) neutered male, 7 (11.3%) intact female, and 25 (40.3%) neutered female dogs (*P* = .91). No sex was given for two dogs with HAC. Dogs with HAC had a median (range) weight of 14.5 kg (2.8 to 52 kg), whereas dogs without HAC weighed 10 kg (2 to 56.3 kg) (*P* = .024).

Common breeds (n ≥ 2) in the HAC group included crossbreed (n = 37), Yorkshire Terrier (n = 10), Dachshund (n = 9), Poodle (n = 6), Beagle (n = 5), Maltese (n = 4), Scottish Terrier (n = 4), Magyar Vizsla (n = 3) and Spaniel (n = 3). There were also two dogs of each of the following breeds: Boxer, Flat‐Coated Retriever, Golden Retriever, Parson Russell Terrier, Giant Schnauzer, Shih Tzu, Whippet. Common breeds (n ≥ 2) in the non‐HAC group included crossbreed (n = 17), Yorkshire Terrier (n = 8), Dachshund (n = 4), Jack Russel Terrier (n = 3), Poodle (n = 3), Shih Tzu(n = 3), and two dogs of each of the following breeds: Beagle, Border Terrier, German Shepherd, Golden Retriever, Maltese, West Highland White Terrier.

Classical clinical signs, such as polyuria (88% vs 52%, *P* < .001), polydipsia (89% vs 58%, *P* < .001), polyphagia (70% vs 31%, *P* < .001), alopecia (52% vs 36%, *P* = .049), lethargy (58% vs 40%, *P* = .034), muscle loss, and/or weakness (36% vs 21%, *P* = .043) were significantly more common in dogs with HAC than in those without HAC, whereas no difference was found in the frequencies of abdominal enlargement (56% vs 47%, *P* = .39), excessive panting (33% vs 26%, *P* = .41), or inappetence (4% vs 5%, *P* = .969).

The final diagnoses recorded (the number of dogs and patterns other than those with complete suppression) in the non‐HAC group were hepatopathy (n = 8; inverse pattern = 1), recurrent dermatitis/pyoderma/folliculitis (n = 7; inverse pattern = 2), diabetes mellitus (n = 6; escape pattern = 1, partial suppression pattern = 1), epilepsy (n = 5), inactive adrenal mass (n = 5; inverse pattern = 3), hypothyroidism (n = 4; escape pattern = 1), cardiopathy (n = 4; escape pattern = 1, partial suppression pattern = 1), central diabetes insipidus (n = 4; inverse pattern = 1), obesity (n = 3; inverse pattern = 1), enteropathy (n = 3), hepatocellular carcinoma (n = 2), nephropathy (n = 2), pheochromocytoma (n = 2), lymphoma (n = 2), cystitis (n = 2), and one each of adenocarcinoma of the perianal glands, hemangiopericytoma, hemangiosarcoma, histiocytoma, Sertoli cell tumor, insulinoma, transitional cell carcinoma of the bladder, renal glucosuria, seasonal alopecia, endometritis, primary polydipsia, laryngeal paralysis, and luxation of the patella (inverse pattern = 1). In 19 patients, two or more diagnoses were documented.

### 3.2 Laboratory and endocrine tests

The ALP and HS‐ALP were measured in 97 (84%) and 80 (70%) dogs with HAC and in 56 (90%) and 44 (71%) dogs without HAC, respectively. The median (range) ALP activities were 477 (12 to 5933) U/L and 353 (1 to 5632) U/l in the HAC group, and 226 (16 to 2694) U/L and 236 (0 to 2350) U/l in the non‐HAC group, respectively. ALP and HS‐ALP were significantly higher in the HAC group dogs (*P* = .001, *P* = .022). The AUC‐ROC of the ALP and the HS‐ALP to discriminate between dogs with and without HAC were 0.656 (95%CI 0.555‐0.758) and 0.626 (0.523‐0.729), respectively.

UCCRs were available in 90 (78%) and 54 (87%) dogs with HAC and without HAC, respectively. The UCCR was significantly higher in dogs with HAC compared to dogs with non‐adrenal illness (*P* = .018) and supportive for HAC (>161.2 × 10^−6^) in 28/90 (31%) of the dogs with later confirmed HAC, respectively. Only two of 54 (4%) dogs without HAC had a UCCR > 161.2 × 10^−6^. Both were Yorkshire Terriers with massive nodular hepatopathy and a negative LDDT and ACTH‐stimulation test. The UCCR was in the diagnostic grey zone between 26.5 and 161.2 × 10^−6^ in 61/90 (68%) and 43/54 (80%) dogs with and without HAC, respectively. One of 90 (1%) and 9/54 (17%) dogs with and without HAC tested negative (<26.5 × 10^−6^), respectively. The one dog with HAC and a negative UCCR was diagnosed with ATH. The area under the curve (AUC) of the ROC curve of the UCCR to discriminate between dogs with and without HAC was 0.769 (95%CI 0.692‐0.847).

Results of the ACTH‐stimulation test were available in 102 (87%) and 47 (76%) dogs with and without HAC, respectively. The post‐ACTH cortisol concentration was ≥579 nmol/L (≥21 µg/dL) in 85/102 (83%) in the HAC group dogs and 5/47 (11%) in the non‐HAC group dogs (*P* < .001). The ACTH‐stimulation test was negative in 13/84 (16%) dogs with PDH, and in 10/14 (71%) dogs with ATH, respectively. One (33.3%) of the three dogs with both PDH and an adrenal tumor and the dog without a differential diagnosis had negative test results. The AUC‐ROC of the ACTH‐stimulation test to discriminate between dogs with and without HAC was 0.861 (95%CI 0.801‐0.921).

For the LDDT, lack of suppression pattern, partial suppression pattern, escape pattern, inverse pattern, and complete suppression pattern was found in 60 (52,2%), 30 (26.1%), 12 (10.4%), 3 (2.6%), and 10 (8.7%) of the dogs with HAC, and in 0 (0%), 2 (3.2%), 2 (3.2%), 8 (12.9%), and 50 (80.7%) of the dogs without HAC, respectively (Figure 1; Table 1). The LRs and PVs of various LDDT patterns and the LDDT, in general, are depicted in Table 1. Due to the poor performance of the inverse LDDT patterns, the calculations of the overall diagnostic performance of the LDDT to detect HAC were repeated without defining this pattern as a positive test. The AUC‐ROC of the LDDT to discriminate between dogs with and without HAC was 0.938 (95%CI 0.902‐0.974). The cutoff associated with the best sensitivity and specificity to differentiate between dogs with and without HAC for the t_8_ cortisol concentration was 31.72 nmol/L (1.15 µg/dL, sensitivity 0.852, specificity 0.984, differential positive rate 0.836, +LR 52.8, −LR 0.13). Eighteen (95%) of 19 dogs with ATH and 2 (66.7%) of the three dogs with PDH and a concurrent adrenal tumor had lack of suppression, whereas one in each group showed partial suppression. The dog with ATH and partial suppression (171‐66‐66 nmol/L [6.2‐2.4‐2.4 µg/dL]) had a right‐sided adrenal mass (1.98 × 1.8 cm) with mineralization, atrophy of the left adrenal gland (4.2 × 1.8 mm), an ACTH concentration below the detection limit of the assay, and a normal pituitary gland on CT. The dog with PDH, an adrenal tumor, and a partial suppression pattern (88‐36‐39 nmol/L [3.2‐1.3‐1.4 µg/dL]) had an invasive adrenal tumor with local thrombus formation in the vena cava, lung metastases, an enlarged contralateral adrenal gland (width 9 mm), an endogenous ACTH concentration of 1.13 pmol/L, and a normal urinary normetanephrine to creatinine ratio (95; upper reference limit 100).

**TABLE 1:** A, Sensitivities, specificities, positive (PPV) and negative (NPV) predictive values, positive (+LR) and negative (−LR) likelihood ratios, and overall accuracies of the low‐dose dexamethasone test (LDDT) regarding the suppression patterns to diagnose hyperadrenocorticism (HAC), and B, regarding the “increasing” pattern to diagnose pituitary‐dependent hyperadrenocorticism (PDH) in dogs with a lack of suppression

A,	HAC (n = 115)	Non‐HAC (n = 62)	Sensitivity (95% CI)	Specificity (95% CI)	PPV (95% CI)	NPV (95% CI)	+LR (95% CI)	−LR (95% CI)	Accuracy (95% CI)
LDDT positive (inverse pattern = positive)	105	12	91.3 (84.6‐95.8)	80.7 (68.6‐89.6)	88.8 (84‐93.6)	83.3 (73.2‐90.1)	4.72 (2.83‐7.87)	0.11 (0.06‐0.2)	87.6 (81.8‐92)
LDDT positive (inverse pattern = negative)	102	4	88.7 (81.5‐93.8)	93.6 (84.3‐98.2)	96.2 (90.8‐98.5)	81.7 (72.7‐88.2)	13.75 (5.32‐35.5)	0.12 (0.07‐0.2)	90.4 (85.1‐94.3)
Lack of suppression pattern	60	0			100			0.48 (0.4‐0.58)	
Partial suppression pattern	30	2			93.8 (78.8‐98.4)		8.09 (2‐32.72)	0.76 (0.68‐0.86)	
Escape pattern	12	2			85.7 (58.1‐96.3)		3.23 (0.75‐14)	0.93 (0.86‐1)	
Inverse pattern	3	8			27.3 (9.4‐57.7)		0.2 (0.06‐0.73)	1.12 (1‐1.2)	
**B,**	**PDH (n = 41)**	**Non‐PDH (n = 18)**			**PPV (95%CI)**		**+LR (95%CI)**	**−LR (95%CI)**	
Increasing pattern	7	2			77.8 (44.6‐93.8)		1.54 (0.35‐6.69)	0.93 (0.75‐1.16)	

The “increasing” pattern was seen in 10 (16.7%) of 60 (41 PDH, 18 ATH, 1 no differentiation) dogs with HAC and the “lack of suppression” pattern. Seven of these dogs were diagnosed with PDH, two with ATH, and no differentiation was possible in one dog (Table 1).

## 4 DISCUSSION

The results of the present study agree with earlier studies assigning a high sensitivity to the LDDT for the diagnosis of HAC,^3^, ^14^, ^15^, ^16^, ^17^, ^18^ As suggested by Bennaim et al, our results confirm that the "inverse pattern" provides no support for HAC.^3^

The sensitivity or true‐positive rate of the LDDT (not including the inverse pattern as a positive test) to diagnose HAC in our patients was 89%, comparable to the 85%‐100% reported in the literature.^3^, ^14^, ^15^, ^16^, ^17^, ^18^ Possible explanations for sensitivities >95% in earlier studies could, in part, be attributed to the different criteria used to confirm the diagnosis. In at least two studies, the LDDT, as a sole test, was used to confirm the diagnosis, which is questionable.^16^, ^17^ In another study, only dogs with a complete necropsy report were included, which likely selected for animals with more severe or advanced disease.^18^ Although the –LR of 0.12 supports an LDDT result that can eliminate an HAC diagnosis in unaffected dogs, the results also show that false‐negative results are possible and that diagnosis should not be based on LDDT‐testing alone. The current recommendations are to perform another test in dogs suspected of HAC with a negative first screening test and rule out HAC only if a second test confirms the negative test result.^1^

Traditionally, a t_4_ cortisol concentration was used to discriminate between PDH and ATH exclusively, whereas the t_8_ concentration was used for both screening and discrimination. Mueller et al described an “inverse” dexamethasone response pattern with a high cortisol concentration at t_4_, but physiologic suppression of cortisol concentrations was seen at t_8_ in 5 (6.25%) of 80 dogs with confirmed HAC.^2^ The authors speculated that the “inverse” pattern might represent a new HAC type and that the t_8_ interpretation might not be accurate. A later retrospective study investigated the diagnostic performance of the LDDT, looking at various patterns in 123 canine patients with suspected HAC and found the “inverse” pattern in 5/123 (4%) dogs.^3^ In that study, the authors recommended using alternative diagnostic criteria for dogs with this specific pattern since there were only 2 (3.4%) and 3 (4.7%) dogs in the HAC‐ and non‐adrenal illness group, respectively, with that pattern. The number of dogs was deemed too small to draw final conclusions. This recommendation is clearly supported by the results of the current study, as the integration of the inverse pattern reduced the +LR and the PPV of the LDDT from 13.75 to 4.72 and from 96.2% to 88.8%, respectively, without a considerable change of the −LR and NPV. LRs, in contrast to PVs, are independent of the disease prevalence and are thought to constitute one of the best ways to measure and express diagnostic test accuracy. The +LR is used as a pre‐test probability multiplier of the respective disease to estimate its post‐test probability. +LRs can range from 0 to infinity, and the higher the number, the more likely the findings suggest the presence of the disease. A +LR >10 depicts substantial changes of post‐test probability estimates and increases the probability of disease by approximately 45%.^19^

The specificity of the LDDT overall, or in other words, the probability of a negative test result in a dog without HAC, was 93% and clearly higher than in all earlier studies. Comparable specificities were found in only one study, where healthy dogs were used as controls.^17^ All other studies reported specificities between 67% and 73%.^3^, ^15^, ^18^ One possible explanation for the high specificity in the present study is the strict exclusion of patients with signs of acute non‐adrenal disease, such as fever, icterus, pain, vomiting, diarrhea, dyspnea, coughing, or untreated diabetes mellitus, as has been strongly recommended.^1^ It is not specified whether the previous studies adhered to these recommendations. Vomiting and diarrhea were reported in 20.3 and 14.6% of the patients in the study of Bennaim et al^3^ It is a well‐known fact that many acute illnesses affect the results of HAC‐screening tests.^1^, ^17^, ^20^ We also excluded patients admitted for the work‐up of obesity, a clinical sign that commonly and wrongly prompts clinicians to test dogs for HAC.^21^ As obesity is extremely prevalent in the general dog population but uncommon in HAC (not including abdominal distension or pendulous abdomen), the inclusion of these dogs would likely have reduced the test accuracy.^21^, ^22^ An additional explanation for the high specificity of the LDDT in this study is the strict avoidance of stress, such as performing diagnostic imaging during the 8‐hour test duration. Mock‐ultrasonography performed during the LDDT raised cortisol concentrations above the diagnostic cutoff in 1 of 6 healthy dogs in one study.^23^ In a recent study, patients were hospitalized for at least one day before the test “to encourage acclimatization and to minimize stress.”^3^ It was shown that single vaccination visits or orthopedic examinations, as well as hospitalizations, increase urinary cortisol excretion^24^; therefore, the advantage of inpatient testing needs further evaluation. In the present study, an individualized approach was chosen, and the dogs were hospitalized only if pet owners considered ambulatory testing more stressful for their animals.

This study clearly confirms that the integration of various suppression patterns can improve the diagnostic performance of the LDDT to “rule in” HAC. Traditionally, only the t_8_ cortisol concentration was used to diagnose HAC. Bennaim et al were the first to examine various LDDT patterns and using PPVs to “rule in” HAC.^3^ In their study, the highest PPVs were observed in dogs with a “lack of suppression” (93.9%), followed by partial suppression (73.1%). The escape (35.7%) and inverse (40%) patterns performed very poorly. The disappointing performance of the “escape” patterns was especially interesting as this is currently considered a classic HAC pattern and was observed in 51 (28%) of 181 dogs with PDH in an earlier study.^4^ The main disadvantage of the PVs is their sensitivity to changes in disease prevalence. Post‐test probabilities, calculated for a referral hospital population, are thus not necessarily valid for patients presented to a primary care practice. To bypass this limitation, we calculated LRs. The results of this study corroborate the superiority of the “lack of suppression” and “partial suppression” patterns. The fact that no (lack of suppression pattern) or very few (3.2% cases with a partial suppression pattern) false‐positive results were observed in dogs with these patterns agrees with the results of Bennaim et al,^3^ who suggested greater hypothalamic‐pituitary‐adrenal axis sensitivity to negative feedback in dogs where HAC was excluded compared with dogs with pituitary and adrenal HAC. The +LRs for the lack of suppression and partial suppression patterns were infinity and 8.09, respectively. A +LR of 8 increases the probability of having the disease by approximately 40% in cases with a positive test results. ^19^ Although the +LR of the “escape pattern” was 3.23, suggesting moderate diagnostic information, the 95%CI was wide and included 1. This result was in line with those of an earlier study^3^ and suggested no association between this pattern and HAC. It is thus advisable to proceed with additional, more specific tests, such as the ACTH‐stimulation test in dogs exhibiting this pattern. Interestingly, a +LR of 0.2 indicated that the “inverse pattern” was a pattern that reduces the likelihood of HAC. An LR < 1 translates to a lower post‐test probability, suggesting that the presence of this pattern reduces the likelihood of HAC. Unfortunately, this specific pattern was observed in 11 dogs only, and the 95% CIs were too wide to allow for reliable, unambiguous assignments.

The second step after diagnosing HAC is to differentiate between PDH and ATH. Any cortisol suppression <50% of basal dexamethasone concentrations or <27.59 nmol/L of the 4‐ or 8‐hour dexamethasone concentrations in a dog with verified HAC, is consistent with a PDH diagnosis.^4^ When applying these cutoffs, approximately 60% of the dogs with HAC can be identified as PDH.^4^ As no differentiation is possible in dogs that show no suppression pattern, other tests, such as the high‐dose dexamethasone test, endogenous ACTH measurement, adrenal ultrasonography, or advanced imaging, are required. In a previous study, an “increasing“ pattern, defined as a >50% increase in cortisol concentrations between any timepoints in dogs without suppression, was observed in 10% of dogs with HAC, and all were diagnosed with PDH.^3^ Consequently, in the present study, we speculated that this new LDDT pattern could support PDH. Although this new pattern was seen in 7 of 41 (17%) dogs that lacked suppression and did not have PDH, it was also observed in 2 of 18 (11%) dogs with ATH. Interestingly, dogs with ATH and a >50% increase in cortisol concentrations during testing had very low basal cortisol concentrations (35.9 and 46.9 mmol/L [1.3 and 1.7 µg/dL]). We hypothesized that the increases observed in these dogs represented short term plasma cortisol concentration fluctuations caused by random ACTH‐independent adrenocortical tumor activity. There were only nine dogs with this pattern in the present study, which was considered a low number. Therefore, the significance of this finding was reduced. However, the +LR near 1 and the 95%CIs that included 1 suggest that an association between the increasing pattern and a pituitary origin for HAC was unlikely.

An increased ALP activity, frequently exceeding 1000 U/L, is the most common biochemical finding in dogs with HAC.^21^ Corticosteroid‐induced ALP is synthesized by the liver after exposure to glucocorticoids and is unique to dogs. This analyte can be easily measured using a routine laboratory procedure due to its heat stability at 65°C.^25^ Although the HS‐ALP analyte is commonly measured in dogs with suspected HAC; its diagnostic value is ambiguous. The fact that the AUC‐ROC of the HS‐ALP and total ALP tests were comparable in this study suggested that the HS‐ALP determination is redundant. Further studies on this topic are in progress.

Besides the retrospective study design and the low number of patients with specific patterns widening the 95% CIs, other limitations of the study must be recognized. First, the final diagnosis of pituitary HAC was based primarily on the response to therapy and was rarely verified by histopathology. Thus, placebo effects^26^ and the fact that most but not all dogs with HAC respond to trilostane^27^ could have impacted the classification of some patients. Additionally, 38 (15.8%) dogs needed to be excluded as criteria, required to justify a final diagnosis, were not fulfilled, and possibly introduced a selection bias and confounded the results. Unfortunately, the low analytic sensitivity of the assay used to measure cortisol inhibited the examination of concentrations <27.59 nmol/L (1 µg/dL) and subsequently the assessment of specific patterns in dogs with low cortisol concentrations.

In conclusion, this retrospective study is in line with earlier studies that assigned LDDT with a good sensitivity for diagnosing HAC in dogs with appropriate clinical signs. The “inverse” pattern decreased the likelihood of HAC, although it remains unclear to what degree, considering the wide 95%CIs in the present study. This study confirmed that the “lack of suppression” and “partial suppression” patterns strongly and moderately supported a diagnosis of HAC, respectively. As no association could be shown between the “escape” pattern and a diagnosis of HAC or non‐adrenal illness, additional diagnostic tests are strongly encouraged in dogs with this pattern. Finally, the low +LR of the recently proposed “increasing” pattern does not support its use as a discriminatory test between PDH and ATH in dogs with HAC.
